# SARS-CoV-2 ORF8: One protein, seemingly one structure, and many functions

**DOI:** 10.3389/fimmu.2022.1035559

**Published:** 2022-10-24

**Authors:** Smita Vinjamuri, Lenong Li, Marlene Bouvier

**Affiliations:** Department of Microbiology and Immunology, University of Illinois at Chicago, College of Medicine, Chicago, IL, United States

**Keywords:** ORF8, SARS-CoV-2, COVID-19, accessory proteins, MHC class I, immune evasion, antigen presentation

## Abstract

SARS-CoV-2 is the virus responsible for the COVID-19 pandemic. The genome of SARS-CoV-2 encodes nine accessory proteins that are involved in host-pathogen interaction. ORF8 is unique among these accessory proteins. SARS-CoV-2 ORF8 shares a surprisingly low amino acid sequence similarity with SARS-COV ORF8 (30%), and it is presumed to have originated from bat. Studies have shown that ORF8 exerts multiple different functions that interfere with host immune responses, including the downregulation of MHC class I molecules. These functions may represent strategies of host immune evasion. The x-ray crystal structure of ORF8 revealed an immunoglobulin-like domain with several distinguishing features. To date, there are numerous unanswered questions about SARS-CoV-2 ORF8 protein and its structure-function relationship that we discuss in this mini-review. A better understanding of how ORF8 interacts with components of the immune system is needed for elucidating COVID-19 pathogenesis and to develop new avenues for the treatment of the disease.

## Introduction

**Figure 1:**
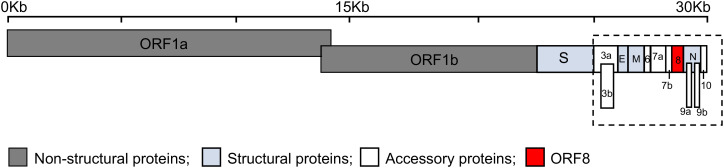
Schematic representation of the genomic organization of SARS-CoV-2. The SARS-CoV-2 genome, ~30 kilobases (Kb), encodes fifteen open reading frames (ORFs) for non-structural (dark grey: ORF1a and ORF1b), structural (light blue: spike (S), envelope (E), membrane (M) and nucleocapsid (N)), and accessory proteins (white: ORF3a, 3b, 6, 7a, 7b, 9a, 9b, and 10) with ORF8 shown in red. The accessory proteins (dashed box) are interspersed among or within the genes encoding structural proteins.

Severe acute respiratory syndrome coronavirus 2 (SARS-CoV-2) is a highly transmissible virus that causes coronavirus disease 2019 (COVID-19), a disease of the lungs (1, 2). SARS-CoV-2 belongs to the genus Betacoronavirus of the family Coronaviridae together with other respiratory viruses such as SARS-CoV and Middle East respiratory syndrome CoV (MERS-CoV) (3). Genomic analyses showed that SARS-CoV-2 shares a high sequence identity with SARS-CoV (79%) and somewhat less with MERS-CoV (50%) (4). The SARS-CoV-2 genome is organized into open reading frames (ORFs) that encode up to twenty nine proteins (**Figure 1**). These twenty nine proteins include four major structural proteins, i.e., spike, envelope, membrane, and nucleocapsid, all of which are required to produce a structurally complete viral particle. Sixteen non-structural proteins (Nsp) are encoded by ORF1a and ORF1b, namely Nsp1-11 and Nsp12-16, respectively. The genes of nine accessory proteins, ORF3a, 3b, 6, 7a, 7b, 8, 9a, 9b, and 10 are interspersed among or within the genes encoding structural proteins (**Figure 1**). Although the accessory proteins are non-essential for viral replication, they have been shown to play a critical role in host-pathogen interaction (5–8). For example, ORF3b, ORF6, ORF7a, and ORF8 act as a type I interferon (IFN) antagonist (9–12). ORF3a, ORF6, ORF7a, and ORF8 interfere with the major histocompatibility complex class I (MHC I) pathway at the transcriptional or post-translational levels (13–17). ORF9b interacts with cellular organelles suppressing antiviral responses, while the functions of ORF7b and ORF10 remain to be further elucidated (8, 18).

There is a particular interest in the ORF8 protein of SARS-CoV-2. Amino acid sequence alignment shows that SARS-CoV-2 ORF8 shares high similarity with Bat-RaTG13-CoV ORF8 (95%) but a rather low homology with SARS-CoV ORF8 (30%), suggesting that its closest relative is Bat-RaTG13-CoV ORF8 (4). The ORF8 gene is part of a hypervariable genomic region that has been recognized as a recombination hotspot, undergoing rapid nucleotide substitutions and deletions (19, 20). One such variant that arose during the early stage of the COVID-19 pandemic has a 382-nucleotide deletion (Δ382) in the ORF7b and ORF8 genes (21). Notably, patients infected with the ORF8 Δ382 variant showed milder COVID-19 symptoms relative to healthy controls. The association of ORF8 deletion with mild COVID-19 prompted a number of studies on characterization of ORF8 function. The results showed that ORF8 exhibits multiple functions directed mainly against host immune responses and affecting different biological pathways. Specifically, it was shown that ORF8 downregulates MHC I (15, 16), antagonizes the IFN signaling pathway (11, 12), interacts with transforming growth factor-beta 2 (22), activates interleukin 17 (IL-17) signaling pathway (23, 24), and induces endoplasmic reticulum (ER) stress (22, 25). The structure of SARS-CoV-2 ORF8 revealed an immunoglobulin (Ig)-like protein with unique characteristics, including the ability to homodimerize (26, 27).

Taken together, SARS-CoV-2 virus is considered a master of immune evasion in causing COVID-19 (28), and ORF8 is viewed as an important virulence factor of SARS-COV-2 pathogenicity and a valuable target of therapeutic development. In this mini-review, we discuss the current knowledge of ORF8 functions with a particular emphasis on those affecting MHC I antigen presentation. We also offer our perspectives on how to reconcile the fact that ORF8, with seemingly one structure, is responsible for a broad range of different cellular functions.

## Modulation of host immune functions

**Figure 2:**
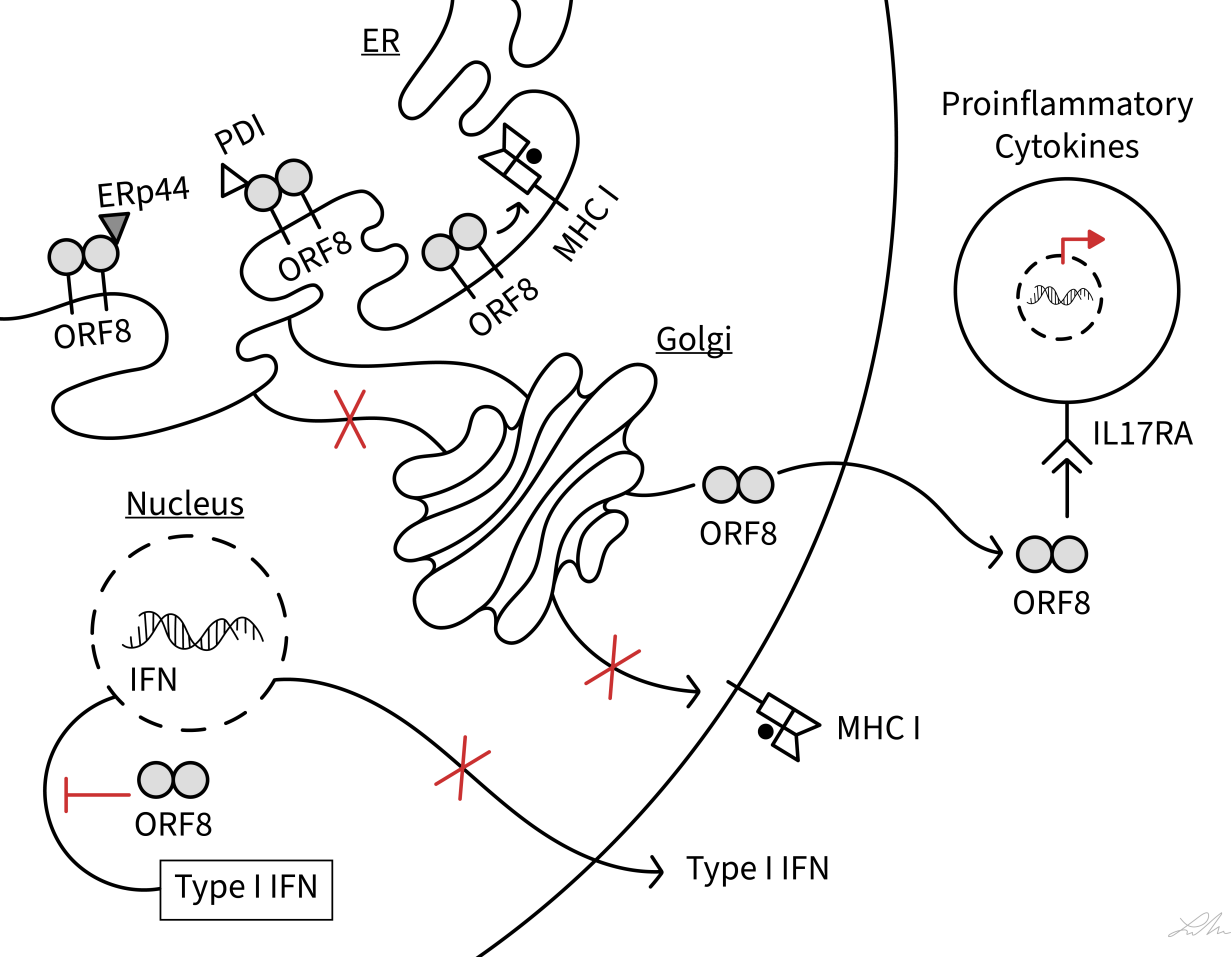
Schematic diagram showing interferences of SARS-CoV-2 ORF8 with various host immune functions. The diagram illustrates the action of ORF8 on MHC class I antigen presentation, ER stress (PDI and ERp44), type I IFN signaling pathway, and IL-17 signaling pathway (IL17RA). See text for ORF8 soluble and membrane-bound status.

COVID-19 patients show a dysregulation of cytotoxic T-lymphocytes (CTLs) and experience a cytokine storm, and these phenotypes have been closely associated with disease severity (29–33). Several of the functions by which ORF8 modulates host immune responses have been characterized (**Figure 2**) and these functions may be central to underlying mechanisms of COVID-19 progression.

### MHC class I antigen presentation

In virus infected host cells, newly synthesized viral proteins are processed by the cytosolic proteasome into small peptides that are then translocated into the ER by the transporter associated with antigen processing (34). In the ER lumen, viral (and endogenous) peptides are selected for loading onto MHC I under the stringent control of a multi-protein complex referred to as the peptide-loading complex (PLC). The PLC generates stable MHC I-peptide complexes which are then transported to the cell surface where the antigenic peptides are presented to CTLs (34). The elimination of virus infected cells critically depends on the detection of viral peptides by CTLs. It was shown that SARS-CoV-2 infection of ACE2-expressing HEK293T cells and lung epithelial cells derived from humanized ACE2 transgenic mice, lead to downregulation of MHC I molecules on infected cells (15). This phenotype was attributed to ORF8 (15, 16). The results also suggested that instead of passing through the Golgi apparatus in route to the cell surface, ORF8 directed the trafficking of MHC I molecules from the ER to lysosomes for degradation *via* the autophagy pathway (15). In this process, ORF8 interacted with Beclin 1, an essential protein for autophagy initiation. Finally, ORF8-mediated interference with the MHC I pathway rendered ORF8-expressing cells and SARS-CoV-2-infected cells less sensitive to lysis by CTLs (15). This study overall was important in demonstrating that ORF8 likely functions as an immune modulator by subverting MHC I antigen presentation, thus enabling SARS-CoV-2-infected cells to evade surveillance by CTLs. To date, however, evidence of a *direct* interaction between ORF8 and MHC I molecules has yet to be provided.

In addition to ORF8, there are at least three additional accessory proteins of SARS-CoV-2 that are capable of interfering with the MHC I pathway: ORF3a, ORF6, and ORF7a. ORF6 was identified as a potential immunomodulatory protein based on its role in targeting NLRC5, a critical transcriptional regulator of the gene coding for MHC I heavy chain (17). More recently, in bioRxiv-deposited articles, ORF3a and ORF7a were shown to reduce MHC I cell-surface expression *via* distinct mechanisms (13, 14). ORF3a exerted a more general effect on suppressing the trafficking of proteins, including MHC I molecules, through the secretory pathway (13). On the other hand, ORF7a acted more specifically by associating with the MHC I heavy chain, possibly as a molecular mimic of β_2_m, thereby slowing the export of correctly assembled MHC I molecules out of the ER (13, 14).

Taken together, SARS-CoV-2 has evolved more than one protein and strategy to interfere with the MHC I pathway and escape immune surveillance from CTLs. This is not surprising given that MHC I-mediated immune responses are central to host defense mechanisms against viral infections. Other human viruses such as cytomegalovirus, Epstein-Barr virus, Kaposi sarcoma-associated herpesvirus, and adenovirus also utilize different strategies to target the MHC I pathway and disrupt antigen presentation (35).

### ER stress

SARS-CoV-2 infection of host cells causes ER stress and activates the unfolded protein response (UPR) (36). The UPR is a host cellular defense mechanism launched in response to misfolded and unfolded proteins that accumulate in the ER to ensure the survival of stressed cells (37). UPR factors work to restore the normal protein folding capacity of the ER and enhance protein output from the ER. Viruses have evolved different strategies to utilize the UPR for viral replication. Interestingly, ORF8 alone can induce ER stress and a molecular mechanism has been proposed (22, 25). It was suggested that ORF8 mimics an unfolded or folding intermediates thereby creating an imbalance in the protein folding milieu of the ER that leads to UPR activation. Under ER stress, it was shown that ORF8 escapes protein degradation by forming mixed disulfide complexes with ER-resident oxidoreductases such as ERp44 and protein disulfide isomerase (PDI) (25). Although ERp57 was not specifically examined in this study, it is a prominent oxidoreductase in the ER (38) and it plays a critical role in MHC I folding and maturation (39, 40). As such, interaction between ORF8 and ERp57, or PDI (25), could contribute to ORF8-mediated impairment of MHC I cell-surface expression.

### IL-17 signaling pathway

A hallmark of severely ill COVID-19 patients is the overproduction of inflammatory cytokines and hyperactivation of immune cells (29–33). Severe COVID-19 disease coincides with elevated serum levels of the proinflammatory cytokine IL-17 (41, 42). Using a yeast two-hybrid system and immunoprecipitation, SARS-COV-2 ORF8 was shown to bind to the receptor of IL-17, namely IL-17 receptor A (IL17RA) (23). This interaction activated IL-17 signaling pathway and promoted secretion of pro-inflammatory factors (23). SARS-COV-2 ORF8 was also shown to bind to IL17RA on the surface of blood monocytes, triggering an inflammatory response stronger than host IL-17 (24, 27). An interaction between ORF8 and IL17RA was also predicted from an analysis of ORF8 interactor networks (43). It was also shown that ORF8 binds to the ectodomain of IL17RA (23), which in itself implies that ORF8 functions as a secreted protein (see also below). These findings overall suggest that ORF8 acts as a potent molecular mimic of host IL-17 to induce IL17RA-mediated inflammation. The modulation of IL-17 signaling pathway by ORF8 is thought to be a critical aspect of SARS-CoV-2-induced inflammation that causes immune cell infiltration and lung injury.

### ORF8 variants

ORF8 exhibits one of the highest levels of sequence variability among the nine accessory proteins of SARS-CoV-2. Several residue substitutions have been identified during the pandemic, with amino acid 84 (Leu/Ser) being dominant natural variants (44, 45). Other common amino acid substitutions are 62 (Val/Leu), 24 (Ser/Leu), and 45 (Trp/Leu) (46, 47). How these variations in amino acids alter ORF8 interaction with host immune components, and whether the variations correlate with enhanced or attenuated ORF8 phenotypes, is mostly uncharacterized. To the best of our knowledge, there are only a few reports in the literature on characterization of ORF8 variants: ORF8Ser84, ORF8Leu62, and ORF8Leu24 variants are associated with milder COVID-19 (48) and they were shown to bind more weakly to blood monocytes (24, 27); and ORF845Leu showed enhanced interactions with interferon regulatory factor 3 (46). A better understanding of how ORF8 variants may confer a functional advantage to ORF8 and SARS-CoV-2 awaits progress in characterization of the molecular and structural basis by which ORF8 interacts with host immune proteins.

## Unique structural features

**Figure 3:**
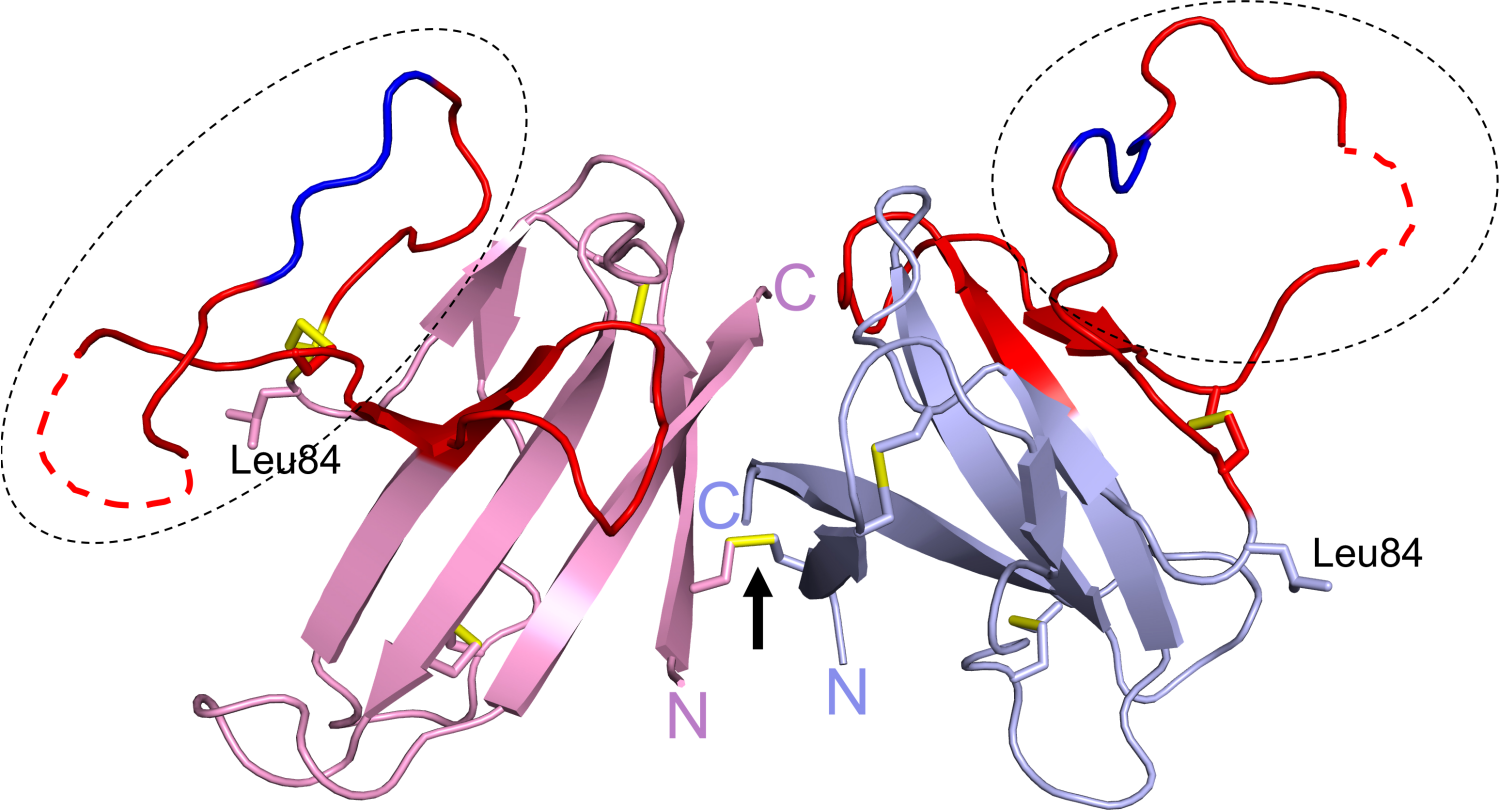
SARS-CoV-2 ORF8 structure. The structure of ORF8Leu84 (PDB code 7JTL) shows a homodimer formed by Cys20-Cys20 disulfide bond (black arrow). Each ORF8 monomer (blue or pink) shows a Ig-like core with a SARS-CoV-2 unique sequence of 38 amino acid residues (red). A large loop (dashed ovals) extends out of the central core; the Y^73^IDI^76^ motif (dark blue) and missing residues ~Glu64 to Ser69 (dashed red lines) are shown. Leu84 and the N- and C-termini are labelled. The three intramolecular and single intermolecular disulfide bonds are shown in yellow.

ORF8 has 121 amino acids consisting of an N-terminal signal sequence (residues 1 to 15) for ER import followed by a central Ig-like domain (residues 16 to 121). The first crystal structure of ORF8 (residues 18 to 121) had Leu84 and revealed a homodimer formed *via* an intermolecular disulfide bond involving Cys20 (**Figure 3**) (26). The central domain of each monomer consists of 7 antiparallel β-strands connected by short loops and stabilized by two intramolecular disulfide bonds (Cys25/Cys90 and Cys37/Cys102). The region between residues Tyr46 to Cys83 (shown in red) represents an insertion specific to SARS-CoV-2 ORF8 and its most recent bat precursors, and it is noticeably lacking in other betacoronavirus proteins with Ig-like folds such as SARS-CoV ORF8ab and SARS-CoV-2 ORF7a (26). This insertion is stabilized by a third intramolecular disulfide bond (Cys61/Cys83) and is also characterized by a prominent loop (residues Asp62 to Asn78) that extends out of the core domain (shown in the dashed ovals). Electron density was missing between ~Glu64 to Ser69 within this loop in each monomer (shown as dashed red lines). The structure of ORF8(Ser84) (residues 16 to 121) has also been reported (27). In contrast to ORF8(Leu84), ORF8(Ser84) crystallized as a monomer that was suggested to potentially form an intermolecular disulfide bond with another molecule of an adjacent symmetric unit (27). ORF8(Ser84) adopts a similar fold as ORF8(Leu84) monomer with differences seen mostly in the loops connecting the β-strands and in the large loop insertion. Both of these ORF8 structures were determined using protein refolded from solubilized bacterial inclusion bodies. Because Cys20 is absent in SARS-CoV ORF8ab, homodimerization is likely a unique phenomenon of SARS-CoV-2 ORF8 and its most recent bat precursors such as RaTG13 ORF8 (26). The structure of RaTG13 ORF8 was also determined and it showed a homodimer formed *via* Cys20 and a similar fold overall as ORF8(Leu84) (27). Another special characteristic of SARS-CoV-2 ORF8 and its closely related bat coronaviruses, is the Y^73^IDI^76^ motif within the loop insertion (shown in dark blue) (**Figure 3**). The structure of SARS-CoV-2 ORF8(Leu84) showed that residues 71 to 75 are involved in a noncovalent dimerization interface between symmetry related molecules in the crystal, with the Y^73^IDI^76^ motif suggested to stabilize this interface (26). The structures of RaTG13 ORF8 supports a similar noncovalent dimer interface in the crystal, while this phenomenon was not observed in the structure of ORF8(Ser84) (note that this variant crystallized with one molecule in an asymmetric unit). Taken together, SARS-CoV-2 ORF8 is distinct from SARS-CoV ORF8ab and other betacoronavirus proteins with Ig-like folds such as SARS-CoV-2 ORF7a, in several ways (1): the presence of a long and conformationally flexible loop that extends out of the Ig-like core domain (2); the ability to form an intermolecular disulfide-linked homodimer *via* Cys20 (3); the propensity to form a noncovalent dimer interface in the crystal *via* the Y^73^IDI^76^ motif that likely supports “oligomerization”.

## Structure-function relationship

It is interesting that an Ig-like protein such as SARS-CoV-2 ORF8 lacks a C-terminus transmembrane region, as seen for example in SARS-CoV ORF8ab and SARS-CoV-2 ORF7a. It was suggested that ORF8 is an extracellularly secreted protein (49) and overexpression of the protein in several cell culture systems such as A549, HEK293T, and BY-2 cells confirmed that ORF8 is secreted as a homodimer (16, 50, 51). A secretory status is consistent with ORF8 eliciting one of the strongest antibody responses among SARS-CoV-2 antigens in infected individuals (52, 53), and with its role as a viral cytokine that binds to IL17RA on monocytes (24, 27). A secretory status, however, raises the intriguing question of how SARS-CoV-2 ORF8 can suppress the cell-surface expression of MHC I (15, 16), and has an interactome that includes predominantly host proteins associated with the ER quality control system (43, 54–56). These characteristics are more consistent with ORF8 accumulating in the ER, and there is mounting experimental evidence consistent with this (15, 16, 25). This in itself is also puzzling given that ORF8 does not harbor an apparent motif for ER retention. It is possible that the N-terminal signal sequences serves as membrane anchoring regions positioning the Ig-like domains of ORF8 homodimer within the ER lumen (49). Alternatively, the ability of ORF8 to form mixed-disulfide bonds with ER-resident oxidoreductases, as part of a mechanism by which ORF8 induces ER stress (22, 25), could play a role in its ER localization. This question remains to be further examined as it is critical to understand the underlying molecular mechanism by which SARS-CoV-2 ORF8 subverts MHC I antigen presentation.

It is also intriguing that multiple distinct host binding partners and functions have been attributed experimentally to SARS-CoV-2 ORF8 (11, 12, 15, 16, 22–25). Interactome studies of ORF8 have also mapped a broad interaction landscape that includes host proteins associated with ER stress and secretory pathways, complement and coagulation cascade, and INF-β signaling pathway (43, 54–56). It is known that some proteins manifest multiple functions through intrinsically disordered regions. Indeed, intrinsically disordered proteins can interact with multiple binding partners and are central to protein interaction networks and for regulating biological mechanisms in cells (57, 58). In line with this general idea, it was suggested that ORF8 may mimic an unfolded protein in cells as a strategy to induce ER stress (25). It was also reported that SARS-CoV-2 ORF8 has a tendency to form aggregates intracellularly when expressed in human lung epithelial cells, and bioinformatics analysis suggested a role for the N-terminal residues 1 to 18 in this phenotype (12). SARS-CoV-2 was also shown to reversibly aggregate with increased in temperatures and at acidic pH, which was different for ORF8(Leu84) (51). Moreover, as discussed above, comparisons of the structures of SARS-CoV-2 ORF8 variants and RatG13 ORF8 showed that the large loop exhibited the most conformational differences (26, 27), and this was further shown by NMR (51). In two of these crystal structures, several residues within this loop lacked clear electron density (**Figure 3**) (26, 27). These observations together highlight the inherent conformational flexibility of the large loop and its potential to adapt in driving productive interaction with various proteins (27). The SARS-CoV-2-specific loop could therefore be an important structural element in endowing ORF8 with the ability to establish interaction networks in cells. It is also noteworthy that SARS-CoV-2 ORF8 was suggested to form a more diverse pattern of intramolecular disulfide bonds in cells than the arrangement seen in its structures (25). The configuration of ORF8 intramolecular disulfide bonds was also suggested to be sensitive to the nature and conformation of the residues adjacent to the Cys residues (27), which notably are often variant sites such as residues 24, 26, 62, and 84. Finally, an *in silico* study indicated that the structure of ORF8, especially the loop regions, was greatly influenced by the status of the intramolecular disulfide bonds (59). The disulfide bonds may therefore represent another structural element associated with conformational adaptability in ORF8. Taken together, the inherent conformational plasticity of the SARS-CoV-2-specific loop, the high number of Cys residues and intramolecular disulfide bonds, the ability to homodimerize, the soluble and/or membrane-bound status, and the potential to form high-order assembly could support ORF8 interaction with different host proteins and, consequently, the manifestation of multiple cellular functions.

## Conclusion

Since the initial isolation of SARS-CoV-2 and characterization of its genome, ORF8 has been recognized as a unique protein. We now have a wealth of new knowledge about ORF8 as discussed in this mini-review, but several important aspects of its properties and structure-function relationship require further investigation. Equally important is the question of whether ORF8 functions alone and/or synergizes with other SARS-CoV-2 proteins in suppressing host immune responses. In conclusion, the ongoing efforts, including our own laboratory, at characterizing ORF8 and the molecular mechanisms by which this accessory protein interferes with host immune functions will eventually generate the knowledge needed to understand how seemingly one structure can support a multi-functional role in cells.

## Author contributions

MB conceptualized the review topic and wrote the manuscript; SV participated in writing and discussion, and drafted **Figure 1**; LL, participated in discussion and made final **Figures 1** and **3**. All authors contributed to the article and approved the submitted version.

## Funding

This work was supported by NIH/NIAID grants R01 AI114467 and R21 AI173863 (to MB).

## Acknowledgments

We thank the researchers worldwide whose work contributed to conceptualize this review topic, even if we did not cite their work. Lauren Muskara from the UIC Biomedical Visualization program is acknowledged for making **Figure 2**.

## Conflict of interest

The authors declare that the research was conducted in the absence of any commercial or financial relationships that could be construed as a potential conflict of interest.

## Publisher’s note

All claims expressed in this article are solely those of the authors and do not necessarily represent those of their affiliated organizations, or those of the publisher, the editors and the reviewers. Any product that may be evaluated in this article, or claim that may be made by its manufacturer, is not guaranteed or endorsed by the publisher.
